# Inhibition of Bcl-2 Sensitizes Mitochondrial Permeability Transition Pore (MPTP) Opening in Ischemia-Damaged Mitochondria

**DOI:** 10.1371/journal.pone.0118834

**Published:** 2015-03-10

**Authors:** Qun Chen, Haishan Xu, Aijun Xu, Thomas Ross, Elizabeth Bowler, Ying Hu, Edward J. Lesnefsky

**Affiliations:** 1 Department of Medicine, Pauley Heart Center, Division of Cardiology, Virginia Commonwealth University, Richmond, Virginia, United States of America; 2 Department of Medicine, Pauley Heart Center, Division of Biochemistry, Virginia Commonwealth University, Richmond, Virginia, United States of America; 3 McGuire Department of Veterans Affairs Medical Center, Richmond, Virginia, United States of America; 4 Department of Anesthesiology, Tongji Hospital, Huazhong University of Science and Technology, Wuhan, China; 5 University of the West of England, Bristol, United Kingdom; Rutgers New Jersey Medical School, UNITED STATES

## Abstract

**Background:**

Mitochondria are critical to cardiac injury during reperfusion as a result of damage sustained during ischemia, including the loss of bcl-2. We asked if bcl-2 depletion not only leads to selective permeation of the outer mitochondrial membrane (MOMP) favoring cytochrome *c* release and programmed cell death, but also favors opening of the mitochondrial permeability transition pore (MPTP). An increase in MPTP susceptibility would support a role for bcl-2 depletion mediated cell death in the calcium overload setting of early reperfusion via MPTP as well as later in reperfusion via MOMP as myocardial calcium content normalizes.

**Methods:**

Calcium retention capacity (CRC) was used to reflect the sensitivity of the MPTP opening in isolated cardiac mitochondria. To study the relationship between bcl-2 inhibition and MPTP opening, mitochondria were incubated with a bcl-2 inhibitor (HA14-1) and CRC measured. The contribution of preserved bcl-2 content to MPTP opening following ischemia-reperfusion was explored using transgenic bcl-2 overexpressed mice.

**Results:**

CRC was decreased in mitochondria following reperfusion compared to ischemia alone, indicating that reperfusion further sensitizes to MPTP opening. Incubation of ischemia-damaged mitochondria with increasing HA14-1concentrations increased calcium-stimulated MPTP opening, supporting that functional inhibition of bcl-2 during simulated reperfusion favors MPTP opening. Moreover, HA14-1 sensitivity was increased by ischemia compared to non-ischemic controls. Overexpression of bcl-2 attenuated MPTP opening in following ischemia-reperfusion. HA14-1 inhibition also increased the permeability of the outer membrane in the absence of exogenous calcium, indicating that bcl-2 inhibition favors MOMP when calcium is low.

**Conclusions:**

The depletion and functional inhibition of bcl-2 contributes to cardiac injury by increasing susceptibility to MPTP opening in high calcium environments and MOMP in the absence of calcium overload. Thus, ischemia-damaged mitochondria with decreased bcl-2 content are susceptible to MPTP opening in early reperfusion and MOMP later in reperfusion when cytosolic calcium has normalized.

## Introduction

Bcl-2 family proteins modulate the propensity of cardiomyocytes to undergo cell death during ischemia and reperfusion [1,2]. These proteins include the anti-apoptotic proteins (bcl-2, bcl-xl, Mcl-1), pro-apoptotic proteins (bax and bak), sensitizer (Bad, Noxa, Puma, Bik, HRF), and direct activators [Bid, truncated bid (t-Bid) and bim]. Pro-apoptotic proteins and activators are usually sequestered by anti-apoptotic proteins [1,2]. Ischemic damage to mitochondria decreases bcl-2 content and also favors opening of the mitochondrial permeability transition pore (MPTP) [3]. Reversible blockade of electron transport during ischemia preserves the bcl-2 content accompanied by decrease in susceptibility to MPTP opening following ischemia. Functional inhibition of the bcl-2 using HA14–1 sensitizes the MPTP opening in cardiac mitochondria from non-ischemic hearts [3]. These results indicate a potential link between decreased bcl-2 content/inhibited bcl-2 function and MPTP opening.

Although strategies applied before ischemia such as ischemic preconditioning [4] and inhibition of mitochondrial respiration [5] provide cardioprotection during ischemia-reperfusion, treatments applied during early reperfusion such as ischemic postconditioning have greater clinical relevance [6]. It is not surprising to observe that strategies applied before ischemia decrease the MPTP opening during reperfusion in that ischemia-mediated mitochondrial damage is prevented by these treatments [7,8]. In contrast, ischemia-mediated mitochondrial damage, including to oxidative phosphorylation, cannot be prevented by interventions applied during early reperfusion [9]. However, interventions applied at the onset of reperfusion still decrease cardiac injury. Ischemic postconditioning attenuates MPTP opening during reperfusion [9,10], suggesting that events during early reperfusion further sensitize to pore opening. Thus, we first asked if the sensitivity to MPTP opening is increased in mitochondria following reperfusion compared to ischemia alone. Inhibition of bcl-2 using HA14–1 increases the MPTP opening in mitochondria from non-ischemic hearts [3]. The bcl-2 content is decreased in cardiac mitochondria following ischemia [3]. We asked if inhibition of the residual bcl-2 in ischemia-damaged mitochondria using HA14–1 further sensitizes to MPTP opening. If a decreased bcl-2 content or functional inhibition contributes a key role in the reperfusion-induced susceptibility to MPTP opening compared to ischemia alone, then overexpression of bcl-2 should inhibit the MPTP opening in cardiac mitochondria following ischemia-reperfusion.

The classic role for antiapoptotic bcl-2 family proteins is the selective inhibition of mitochondrial outer membrane permeabilization (MOMP) [11]. The opening of MPTP increases the permeability of the inner mitochondrial membrane that leads to mitochondrial swelling and subsequent rupture of the outer mitochondrial membrane. In contrast, MOMP selectively affects the integrity of the outer mitochondrial membrane. The decreased bcl-2 content during ischemia favors the unopposed action of pro-death activator peptides to access and activate bax and bak leading to increased permeability of the outer membrane [1,12,13]. Although the administration of cyclosporine A inhibits the MPTP opening in the buffer perfused heart, cyclosporine A does not prevent bax relocation and insertion into mitochondria [14]. This result suggests that MOMP can occur even after the MPTP is already closed or has been blocked. Calcium overload [15] accompanied by oxidative stress is a key factor for the induction of MPTP during early reperfusion [8,16]. We investigated if functional inhibition of bcl-2 in the ischemia-damaged mitochondria in the absence of calcium increases MOMP. The loss of proteins located within the mitochondrial intermembrane space was used as a bcl-2 dependent indicator of MOMP. The present study found that ischemia-damaged mitochondria with electron transport chain induced depletion of bcl-2 [3] can mediate cardiomyocyte cell death during reperfusion by reinforcing mechanisms and timing. First, in the calcium overload setting of early reperfusion, these mitochondria have enhanced susceptibility to MPTP opening, followed by, even if calcium content recovers in still viable myocytes, the propensity to activate cell death via MOMP and cytochrome *c* release in low exogenous calcium settings.

## Methods

### Isolated cardiac mitochondria from the isolated hearts

The Animal Care and Use Committees of the McGuire VA Medical Center and Virginia Commonwealth University approved the protocol. The isolated rabbit heart [17] was used as the model of ischemia in order to have larger amounts of SSM available for the studies of HA14–1 incubation. Briefly, New Zealand White Rabbits (2–4 kg) were anesthetized with pentobarbital sodium (65 mg/kg iv), and the hearts were isolated and subjected to 30 min no-flow global ischemia at 37°C. Non-ischemic hearts (0 min. ischemia) were used as control. Subsarcolemmal mitochondria (SSM) were isolated according to Palmer et al. [18], with minor modifications as previously published [19]. Hearts (control or hearts following global ischemia) were placed into Chappel-Perry buffer at 4°C, finely minced and homogenized with a polytron tissue processor (Brinkman Instruments, Westbury, NY) for 2.5 seconds at a rheostat setting of 3.5 in Chappel-Perry buffer containing 0.2% bovine serum albumin. SSM were isolated following differential centrifugation [19]. Oxygen consumption in mitochondria was measured using a Clark-type oxygen electrode at 30°C using glutamate (complex I substrate), succinate + rotenone (complex II) and TMPD-ascorbate plus rotenone (complex IV substrate) as donors to specific sites in the electron transport chain [19].

### Determination of calcium retention capacity (CRC)

Mitochondrial tolerance to calcium loading was studied in the single cell fluorimeter (LS-55, PerkinElmer, Waltham MA) using repetitive calcium pulses [10]. Freshly isolated SSM (0.4 mg/ml) were incubated in buffer (150 mM sucrose, 50 mM KCl, 2 mM KH_2_PO_4_, and 20 mM Tris/HCl, pH 7.4) for 90 sec with stirring at 30°C with 0.5 uM calcium green. Succinate (5 mM) was used as substrate. Pulses of calcium (20 nmoles) were added at 60 sec intervals. The number of pulses that resulted in a rapid, irreversible calcium release, indicating onset of MPTP, was recorded.

### Incubation of isolated SSM *in vitro*

Isolated rabbit SSM (1 mg/ml) were incubated in buffer (composition, in mM: sucrose 200, MOPS 25, and mannitol, 50) for 30 min at 30°C in the presence or absence of HA14–1 when succinate (5 mM) was used to energize the mitochondria. Since HA14–1 was first dissolved in DMSO, the same concentration of DMSO was used as vehicle control. At the end of incubation, samples were centrifuged at 20,000 g to separate mitochondria and supernatant. Then, mitochondria and supernatant were dissolved in sample buffer and heated at 95°C for 5 min for immunoblotting.

### Immunoblotting analysis

Equal amounts of protein were loaded onto 4–15% or 4–20% SDS-PAGE, electrophoresed and transferred to a PVDF membrane. The membranes were first blotted by 5% non-fat milk for one hour followed by exposure to primary antibodies overnight. Antibodies (bcl-2, AIF. and subunit IV of cytochrome oxidase) were purchased from Cell Signaling Technology (Danvers, MA). AK-2, bax, and bid antibodies were purchased from Santa Cruz (Santa Cruz, CA). Cytochrome *c* antibody was purchased from BD Science (San Diego, CA). The blots were incubated with peroxidase conjugated anti-rabbit or anti-mouse secondary antibody for 1 hour prior to ECL detection. The intensities of blotting were quantified by densitometry.

### Preparation of mouse hearts for perfusion

Wild type and bcl-2 overexpressed mice, a generous gift from Dr. Balvin Chua [20], were bred in the McGuire VA Medical Center Animal Facility. Mice (2–3 month old) were anesthetized with sodium pentobarbital (100 mg/kg i.p.) and anti-coagulated with heparin (1000 IU/kg i.p.). Hearts were excised and retrograde perfused via the aorta at a constant pressure of 100 mm Hg, with modified Krebs-Henseleit buffer (pH 7.35–7.45 at 37°C) containing 115 mM NaCl, 4 mM KCl, 1.2 mM MgSO_4_, 0.9 mM KH_2_PO_4_, 22.5 mM NaHCO_3_, 2.0 mM CaCl_2_, and 5.5 mM glucose, and oxygenated with 95% O_2_/5% CO_2_. Left ventricular developed pressure was measured with a balloon inserted into the left ventricle. Hearts were equilibrated for 15 min followed by 40 min stop-flow global ischemia at 37°C and subsequent 30 min reperfusion. Hearts were harvested for mitochondrial isolation at the end of the experiment. SSM were isolated as described above.

### Statistical Analysis

Data are expressed as the mean ± standard error of the mean. Differences between different dose treatments including CRC and cytochrome *c* content were compared by one-way analysis of variance with post hoc comparisons performed using the Student-Newman-Keuls test of multiple comparisons. Unpaired student t-test was used to evaluate the differences between two groups. A difference of p<0.05 was considered significant (SigmaStat for Windows Version 1.0, Jandel Corporation).

## Results

### Bcl-2 inhibition sensitized the MPTP opening in ischemia-damaged SSM

**Fig 1:**
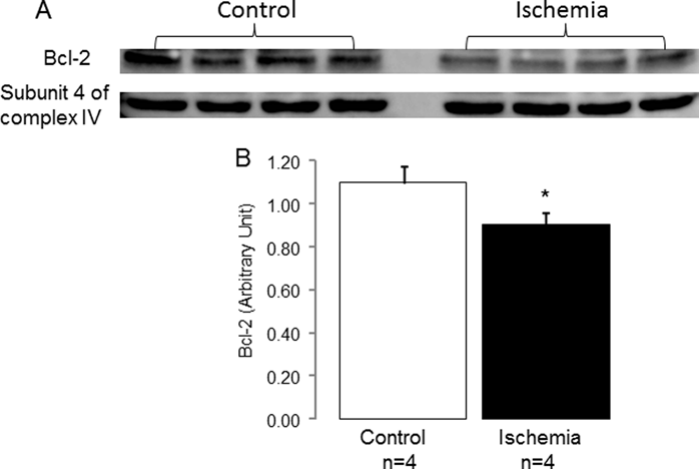
Ischemia decreased bcl-2 content in rabbit SSM. Panel A: Western blotting shows that ischemia markedly decreased bcl-2 content in SSM isolated from rabbit heart compared to non-ischemic hearts. The subunit 4 of cytochrome oxidase was used as protein loading control. Panel B: Ratio of the intensity of bcl-2 over the intensity of subunit 4 of cytochrome oxidase was decreased in ischemia-damaged SSM compared to control (Mean ± SEM; *p<0.05 vs. control).

**Fig 2:**
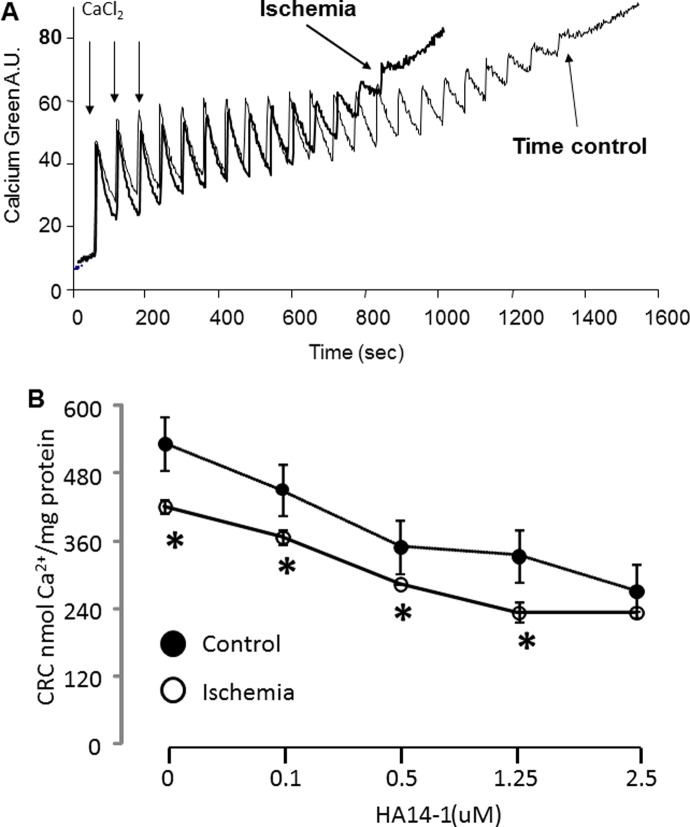
Inhibition of bcl-2 decreases the CRC in ischemia-damaged mitochondria. Panel A: A representative original tracing of the CRC measurement. The pulses of calcium that required for MPTP opening are significantly decreased in ischemia-damaged SSM (subsarcolemmal mitochondria) compared to control. Panel B: Titration with HA14–1 decreases the CRC in both control and ischemic mitochondria in a dose-dependent manner. The concentration of HA14–1 required to reach maximal MPTP opening in ischemia-damaged SSM are lower than that in control SSM, indicating that the sensitivity to HA14–1 inhibition is increased in the ischemic damaged SSM compared to control (Mean ± SEM; *p<0.05 vs. corresponding control).

SSM isolated from control rabbit hearts were well coupled (S1 Table). Ischemia led to decreased state 3 respiration using both complex I and complex II substrates (S1 Table), consistent with our previous findings [3,19]. Ischemia also resulted in a decrease in bcl-2 content in rabbit SSM (Fig. 1). In the isolated rabbit SSM, ischemia decreased the CRC compared to time control (Fig. 2). Titration of HA14–1 decreased the CRC in a dose-dependent manner in non-ischemic control SSM, supporting that functional inhibition of bcl-2 increased the susceptibility to MPTP opening. The CRC was decreased to a greater extent in HA14–1 treated SSM from ischemic hearts at any given dose of HA14–1. Furthermore, a lower concentration of HA14–1 was required to reach the maximal decrease in CRC, indicating an enhanced sensitivity of mitochondria following ischemia to functional inhibition of the remaining bcl-2. In SSM from ischemic hearts, the CRC was decreased to lowest value at 1.25 uM HA14–1 whereas 2.5 uM HA14–1 was required to achieve the lowest CRC value in non-ischemic SSM. In ischemia damaged SSM, it is likely that the decrease in CRC without addition of HA14–1 is due in part to the depletion of bcl-2 by ischemia (Fig. 1), with the lower concentrations of HA14–1 required to achieve a lower CRC values throughout the concentration-response curve. The lower dose of HA14–1 required to achieve decreases in CRC is due in part to greater relative inhibition of the decreased residual content of bcl-2. As a positive control, a high dose of HA14–1 resulted in maximal sensitivity to MPTP with similar CRC in control SSM and ischemic SSM, demonstrating in the presence of the high dose of HA14–1 that a saturated sequestration of bcl-2 led to a similar degree of the MPTP opening in control and ischemic SSM, suggesting that a major contribution to MPTP sensitivity by bcl-2. This finding further supports the importance of bcl-2 depletion in enhancing sensitivity to MPTP during reperfusion.

### Inhibition of bcl-2 increased release of cytochrome c from ischemia-damaged SSM in the absence of calcium as an indicator of MOMP

**Fig 3:**
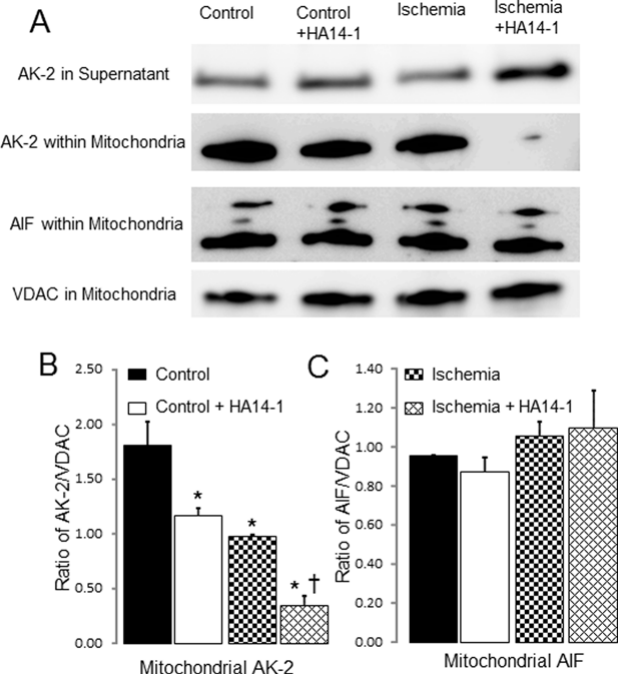
Inhibition of bcl-2 increases the release of AK-2 from ischemia-damaged mitochondria. In control mitochondria, HA14–1 treatment led to compared to no HA14–1 treated mitochondria (Panel A &B). The AK-2 content was markedly decreased in the ischemia-damaged SSM compared to control (Panel B). HA14–1 treatment further decreased the AK-2 content in ischemia-damaged SSM with corresponding increase in supernatant compared to ischemia alone SSM (Panel A & B). HA14–1 inhibition or ischemia did not lead to the release of apoptosis inducing factor (AIF) from mitochondria (Panel C). Voltage dependent anion channel (VDAC) is used as the protein loading control.

First we tested if HA14–1 treatment increased the permeability of the outer mitochondrial membrane. AK2 (Adenylate kinase 2) is an unbound protein free in the intermembrane space. In control SSM, HA14–1 treatment depleted AK2 within mitochondria and correspondingly increased AK2 content in supernatant, supporting that functional inhibition of bcl-2 increased permeation of the outer mitochondrial membrane that led to a release of AK2 from mitochondria into supernatant. HA14–1 treatment also induced the AK2 release from mitochondria to supernatant in ischemia-damaged mitochondria. However, the content of AK2 released from ischemic SSM was less than that in control SSM, suggesting that some of AK2 in ischemic SSM were already released from mitochondria into cytosol during in situ ischemia, indicating that ischemia results in an increase in the permeation of the outer membrane that led to a portion of AK2 loss (Fig. 3).

**Fig 4:**
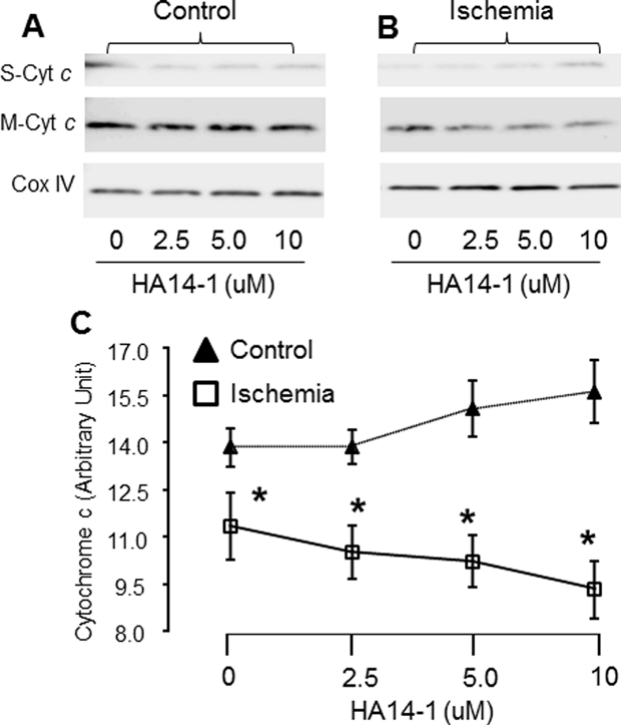
The effect of bcl-2 inhibition on cytochrome *c* content in control and ischemia-damaged mitochondria. Panel A: A representative image shows that titration of HA14–1 leads to decreased cytochrome *c* content in ischemia-damaged SSM with a corresponding increase in supernatant in a dose-dependent manner. Panel B: The ratio of intensity of cytochrome *c* to the intensity of subunit 4 of cytochrome oxidase. The decreased cytochrome *c* content in ischemia-damaged mitochondria compared to control in the absence of HA14–1, suggests that ischemia already leads to partial cytochrome *c* loss before mitochondria are isolated from ischemic hearts. The further decrease in cytochrome *c* content in HA14–1 treated ischemic mitochondria indicates that inhibition of bcl-2 can lead to further release of cytochrome *c* into supernatant (Mean±SEM; *p<0.05 vs. corresponding control).

Cytochrome *c* is also an intermembrane space protein tightly associated with cardiolipin, a key lipid component of the inner mitochondrial membrane [21]. The content of cytochrome *c* was decreased in SSM following ischemia compared to controls in the absence of HA14–1 treatment (Fig. 4), supporting the notion that ischemia led to a partial release of cytochrome *c* from mitochondria as previously described [19]. Titration of HA14–1 did not increase cytochrome *c* release in control SSM, suggesting that inhibition of bcl-2 alone is not sufficient to mobilize cytochrome *c* from the inner membrane. HA14–1 treatment increased the loss of cytochrome *c* from ischemia-damaged SSM accompanied by an increase in cytochrome *c* content in supernatant in a dose-dependent manner, indicating that functional inhibition of bcl-2 increases the permeability of the outer mitochondrial membrane that allows the release of cytochrome *c* (Fig. 4), likely following mobilization from the inner membrane as a result of the previously described cardiolipin depletion that occurs during ischemia [22].

AIF (apoptosis inducing factor) is another protein located within the mitochondrial intermembrane space. In contrast to AK2, AIF is anchored on the inner membrane. AIF must be delocalized from the inner membrane by activated mitochondrial calpain before it can be released from mitochondria [23,24]. Ischemia did not alter AIF content in SSM compared to control (Fig. 3), supporting the notion that the loss of AIF from mitochondria occurs during reperfusion rather than during ischemia [25]. HA14–1 treatment also did not decrease AIF content in either control or ischemic SSM, supporting that selective permeation of the outer membrane alone is not sufficient to induce AIF loss from mitochondria (Fig. 3).

### Overexpression of bcl-2 improved the CRC in mouse SSM following ischemia-reperfusion

**Fig 5:**
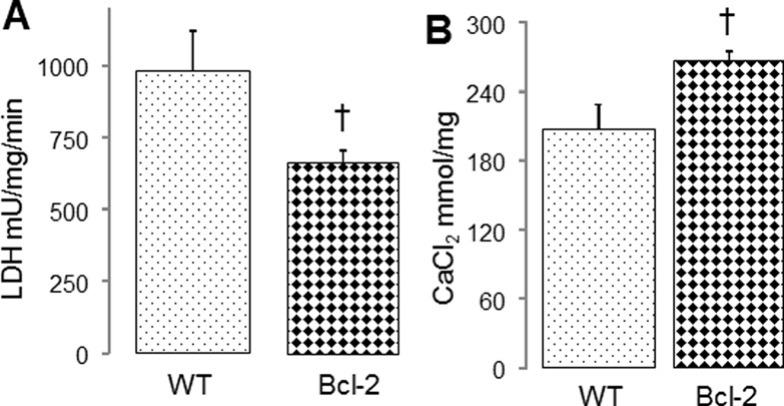
Overexpression of bcl-2 improves the CRC in mitochondria following ischemia-reperfusion. Panel A: The LDH content in coronary effluent in bcl-2 overexpressed mice following 40 min global ischemia and 30 min reperfusion was much lower than that in wild type, supporting that overexpression of bcl-2 reduces cell injury during ischemia-reperfusion. Panel B: Overexpression of bcl-2 improves the CRC in mouse SSM following ischemia-reperfusion compared to wild type, indicating that the sensitivity to MPTP opening is decreased in mitochondria from bcl-2 overexpressed mouse heart. (Mean ± SEM; *p<0.05 vs. pre-ischemia; **†** p<0.05 vs. wild type; n = 6 in each group).

Overexpression of bcl-2 decreases cardiac injury in buffer perfused mouse hearts [20]. Bcl-2 content was markedly increased in both cytosol and SSM from bcl-2 overexpressed heart compared to wild type (S1 Fig.). In the present study, the LDH content in coronary effluent in bcl-2 overexpressed mice was much lower than that in wild type following ischemia-reperfusion (Fig. 5, Panel A), supporting the idea that overexpression of bcl-2 led to decreased cardiac injury. The CRC was improved in mitochondria from bcl-2 overexpressed mouse hearts following reperfusion compared to wild type (Fig. 5, Panel B). Thus, a preserved content of bcl-2 during early reperfusion attenuated the susceptibility to MPTP.

### Reperfusion augmented the MPTP opening in the isolated SSM

Buffer perfused rat hearts were subjected to 25 min global ischemia alone or 25 min ischemia + 30 min reperfusion. Ischemia-reperfusion led to increased LVEDP and decreased LVDP compared to before ischemia (S2A&B Fig.). Ischemia decreased the maximal rate of respiration in SSM isolated from buffer perfused rat hearts compared to time control perfusion. Reperfusion did not further decrease the rate of oxidative phosphorylation compared to ischemia-only SSM (S2C Fig.). Mitochondria isolated from time control hearts were well coupled with RCR value > 9 (S2 Table). Ischemia or ischemia-reperfusion markedly decreased the RCR (S2 Table). Consistent with our previous work, ischemia-damaged SSM showed a decreased CRC compared to time control (S2 Fig., Panel D). The CRC was further decreased following reperfusion compared to ischemia-only SSM (S1 Fig., Panel D), supporting that reperfusion further sensitizes to MPTP opening. Bcl-2 content is also decreased in heart cytosol (S3 Fig.) and mitochondria (S4 Fig.) following reperfusion compared to time control, indicating that the decrease in bcl-2 content persisted during reperfusion.

## Discussion

Ischemic damage of the ETC increases the susceptibility to opening of MPTP during re-oxygenation [3]. Reperfusion does not further damage the electron transport chain nor further decrease respiration [17], but it sensitizes to MPTP opening compared to mitochondria only subjected to ischemia [9,10]. In the present study, titration of HA14–1 further sensitizes to mitochondrial MPTP opening in ischemia-damaged SSM, suggesting that further functional bcl-2 inhibition increases MPTP opening in ischemia-damaged mitochondria. Overexpression of bcl-2 decreases the susceptibility to MPTP opening in buffer perfused hearts following reperfusion supporting, the idea that depletion or inhibition of bcl-2 during ischemia-reperfusion sensitizes mitochondria to MPTP opening. Incubation of HA14–1 with ischemia-damaged SSM in the absence of calcium leads to loss of cytochrome *c* from mitochondria, indicating that functional inhibition of bcl-2 in the absence of calcium increases the selective permeation of the outer membrane (MOMP) during reperfusion. Thus, depletion and inhibition of bcl-2 in ischemia-damaged mitochondria increases cardiac cell death through mechanisms dependent on the calcium environment of the mitochondria; an increase in MPTP opening in high calcium settings and via MOMP in low calcium environments.

Although ischemia damages the ETC in heart mitochondria, the MPTP rarely opens during ischemia in situ in that ischemia-mediated intracellular acidification prevents the MPTP opening [7,8]. MPTP opening is favored in situ during reperfusion when intracellular pH is normalized and calcium overload with ROS generation occurs [7,8]. In the current study, MPTP opening is assessed in normal pH buffer with calcium stimulation in mitochondria following ischemia alone or after reperfusion. The CRC is further decreased in mitochondria following reperfusion compared to ischemia alone, indicating that early reperfusion in situ further sensitizes to MPTP opening. Since the CRC is examined under identical experimental conditions, additional events occurred in situ during early reperfusion to contribute to the further increase in susceptibility to MPTP opening.

Oxidative stress is one key factor that increases MPTP opening during reperfusion [7,8]. ROS production is increased in heart mitochondria isolated following both ischemia alone as well as after reperfusion [26,27]. Compared to ischemia-damaged mitochondria, reperfusion does not further increase the net release of H_2_O_2_ [26,28]. Thus, the generation of ROS alone is not sufficient to explain the increased sensitivity to MPTP opening observed in mitochondria after reperfusion.

Decreased bcl-2 content is associated with increased MPTP opening in ischemia-damaged SSM [3]. HA14–1 inhibition increases the calcium-stimulated MPTP opening in both non-ischemic and ischemia-damaged mitochondria, but the ischemia-damaged SSM appear more sensitive to HA14–1 inhibitor treatment than non-ischemic control mitochondria. The decreased bcl-2 content in the ischemia-damaged mitochondria should require less HA14–1 to block the function of the remaining bcl-2. Thus, functional inhibition of the residual bcl-2 in ischemia-damaged mitochondria sensitizes mitochondria to MPTP opening likely during early reperfusion when calcium over load and oxidant burst interact with the electron transport chain-induced decrease in bcl-2 to predispose to MPTP. Ischemia-reperfusion increases the formation of activators (Bid, t-Bid, Bim) and sensitizer (Bad, Noxa, Puma, Bik, HRF) peptides in buffer-perfused hearts [11,29]. The decreased bcl-2 content after ischemia favors sequestration of the residual bcl-2 in ischemia-damaged SSM by these activators and sensitizers leading to sensitization to MPTP opening during early reperfusion. We attempted to employ an analytical approach to assess the interaction of bcl-2 with sensitizer and activator peptides in response to ischemia and/or reperfusion using co-immunoprecipitation, but this approach proved to be experimentally challenging. Thus, the current approach to assess for the contribution of functional inhibition of bcl-2 was employed.

Calcium overload is another critical factor to induce MPTP during reperfusion [8,16]. The mitochondrial calcium overload gradually increases during ischemia and peaks at early reperfusion [30]. Additional calcium overload within mitochondria may facilitate the MPTP opening following reperfusion compared to ischemia. Thus, the decreased bcl-2 content in combination with additional calcium overload stress and oxidative damage [31] may sensitize the MPTP opening in mitochondria following reperfusion compared to mitochondria following ischemia alone.

Calcium-stimulated MPTP is increased in the presence of bcl-2 inhibitor during oxidative stress [3,32]. However, surviving myocytes regulate intracellular calcium during reperfusion [16]. In the present study, inhibition of bcl-2 using HA14–1 also leads to the cytochrome *c* loss from mitochondria in the absence of calcium overload. The MPTP is unlikely to be opened in incubation buffer without calcium. These results indicate that in the absence of calcium overload, the inhibition of bcl-2 increases MOMP. Thus, functional inhibition of bcl-2 increases the MOMP in post-ischemic SSM, indicating that the imbalance between anti-apoptotic proteins and pro-apoptotic proteins can increase mitochondrial-driven cardiomyocyte injury via MOMP. Intra-cellular calcium overload starts during ischemia and further increases during earlier reperfusion. Intracellular calcium concentration gradually decreases during late reperfusion. In contrast, much of the programmed cell death occurs during late reperfusion [30]. The decreased bcl-2 content persists into the period of reperfusion (S4 Fig.) when calcium normalizes after 30–60 min reperfusion in surviving cells [30]. The imbalance between anti-apoptotic proteins and pro-apoptotic proteins likely contributes to mitochondrial and cell injury during later reperfusion when intra-cellular calcium has already decreased toward normal levels.

The persistent low level of bcl-2 in cardiac mitochondria during the initial course of reperfusion can increase cardiac injury by sensitizing to MPTP opening. The decreased bcl-2 content results in an imbalance between anti-apoptotic proteins and pro-apoptotic proteins on the mitochondrial outer membrane that continues to augment mitochondrial and myocardial injury via MOMP even in the absence of calcium overload during later reperfusion. Thus, the electron transport chain driven decrease in bcl-2 content [3] not only affects the acute injury during early reperfusion but also likely contributes to continued cardiomyocyte loss as reperfusion continues, predisposing toward heart failure during later periods of attempted recovery.

## Supporting Information

S1 Fig The bcl-2 content in cytosol and mitochondria from wild type and bcl-2 over expressed mice. The cytosol and SSM were isolated from wild type and bcl-2 overexpressed mouse heart. The bcl-2 content was determined using western blotting. As expected, the bcl-2 content was markedly increased in both cytosol and SSM from bcl-2 overexpressed heart compared to wild type. GAPDH was used as cytosolic protein loading control. VDAC was used as mitochondrial protein loading control.
(TIF)

S2 Fig Ischemia-reperfusion leads to decreased CRC in mitochondria isolated from buffer-perfused hearts. Male Fisher 344 rats (6–8 month old) were anesthetized with sodium pentobarbital (100 mg/kg i.p.) and anti-coagulated with heparin (1000 IU/kg i.p.). Hearts were excised and retrograde perfused via the aorta at a constant pressure of 100 mm Hg, with modified Krebs-Henseleit buffer (pH 7.35–7.45 at 37°C) containing 115 mM NaCl, 4 mM KCl, 1.2 mM MgSO4, 0.9 mM KH2PO4, 22.5 mM NaHCO3, 2.5 mM CaCl2, and 5.5 mM glucose, and oxygenated with 95% O_2_/5% CO_2_. Left ventricular developed pressure was measured with a balloon inserted into the left ventricle. Hearts were allocated into three experimental groups: Time control, hearts were buffer perfused for 40 min without ischemia; Ischemia, hearts were equilibrated for 15 min followed by 25 min stop-flow global ischemia at 37°C; Ischemia-reperfusion, hearts were equilibrated for 15 min followed by 25 min stop-flow global ischemia at 37°C and 30 min reperfusion. Panel A: There are no differences in left ventricular developed pressure (LVDP) before ischemia among groups. The LVDP is significantly decreased in hearts following ischemia-reperfusion. Panel B: There are no differences in left ventricular end diastolic pressure (LVEDP) before ischemia among groups, but the LVEDP is elevated at the end of ischemia and during reperfusion. Panel C: The rate of oxidative phosphorylation is decreased in mitochondria following ischemia or ischemia-reperfusion compared to time control when glutamate, succinate, and TMPD-ascorbate are used as complex I, II, and IV substrates, respectively. Panel D: Ischemia decreased CRC compared to time control. Reperfusion further decreased the CRC compared to ischemia, indicating that reperfusion further sensitizes the MPTP opening (Mean ± SEM; *p<0.05 vs. time control; **†** p<0.05 vs. ischemia).
(TIF)

S3 Fig Ischemia-reperfusion decreased cytosolic bcl-2 content. Buffer perfused rat hearts were subjected to 25 min global ischemia and 30 min reperfusion. Bcl-2 content in cytosol was determined using western blotting. The bcl-2 content is decreased in hearts following ischemia-reperfusion compared to time control. Tubulin was used as a protein loading control.
(TIF)

S4 Fig Ischemia-reperfusion decreased bcl-2 content in rat. Rat SSM were isolated at the end of heart perfusion. Bcl-2 content was determined using western blotting. Panels A and B: The bcl-2 content is decreased in rat SSM following reperfusion compared to time control (Mean ± SEM; *p<0.05 vs. non-ischemic control).
(TIF)

S1 Table The alteration of oxidative phosphorylation in buffer perfused rat hearts following ischemia (ISC) and reperfusion (REP). (DOCX)

S2 Table Ischemia (ISC) alone leads to decreased rate of oxidative phosphorylation in rabbit heart mitochondria. (DOCX)
