# Supplementary figures and images for: Inhibition of Bcl-2 Sensitizes Mitochondrial Permeability Transition Pore (MPTP) Opening in Ischemia-Damaged Mitochondria
Source: PLoS One. 2015 Mar 10;10(3):e0118834. doi: 10.1371/journal.pone.0118834 (PMC4354902; doi:10.1371/journal.pone.0118834)

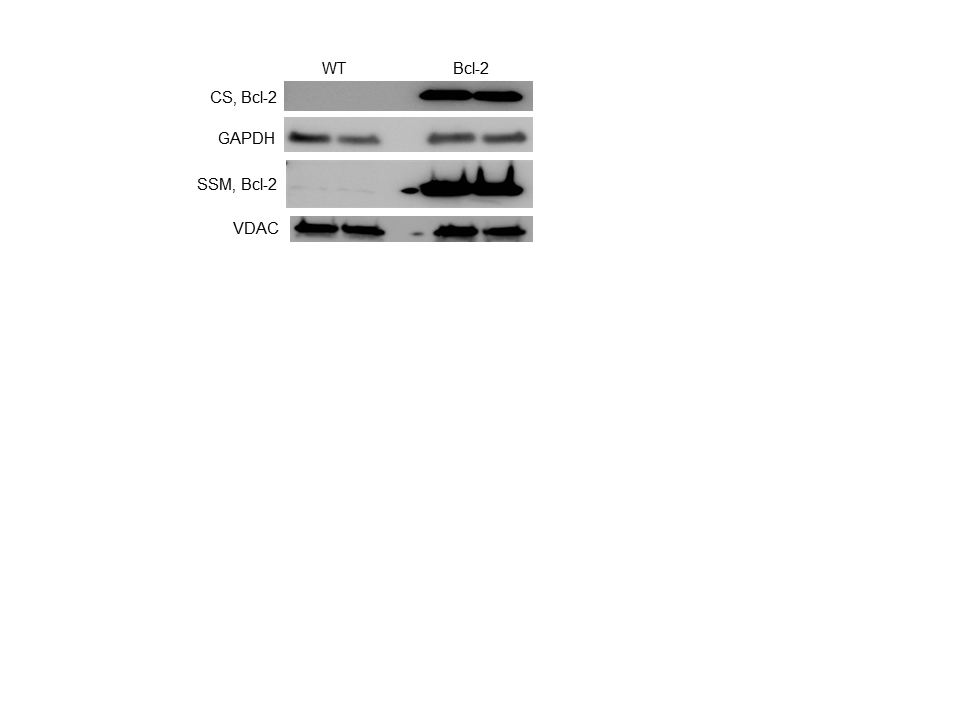

Supplement: S1 Fig — The cytosol and SSM were isolated from wild type and bcl-2 overexpressed mouse heart. The bcl-2 content was determined using western blotting. As expected, the bcl-2 content was markedly increased in both cytosol and SSM from bcl-2 overexpressed heart compared to wild type. GAPDH was used as cytosolic protein loading control. VDAC was used as mitochondrial protein loading control. (TIF) [file pone.0118834.s001.tif]

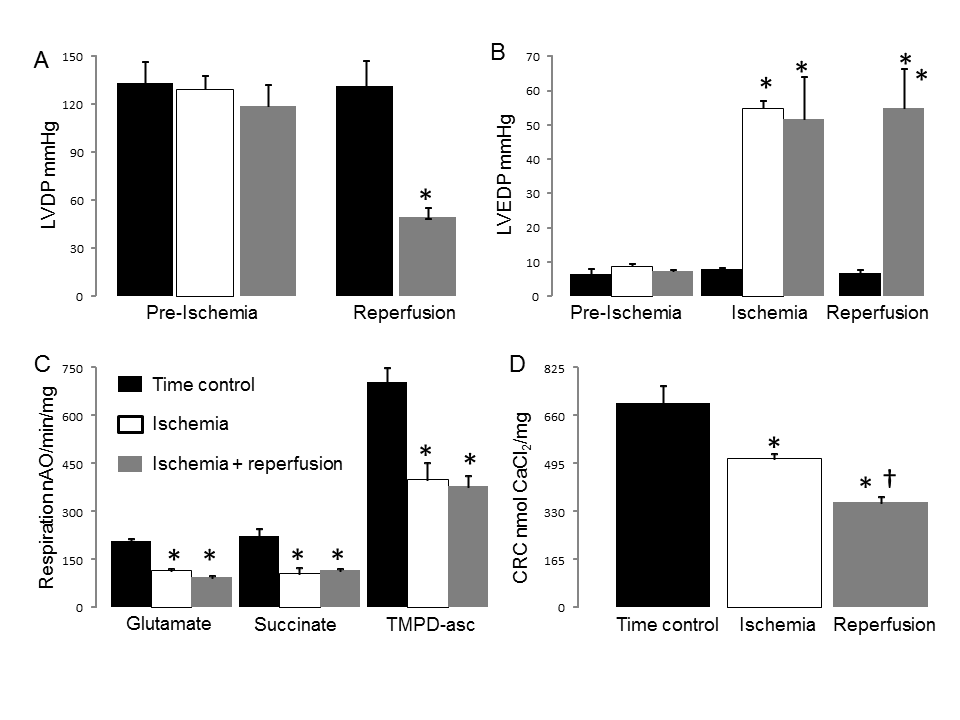

Supplement: S2 Fig — Male Fisher 344 rats (6–8 month old) were anesthetized with sodium pentobarbital (100 mg/kg i.p.) and anti-coagulated with heparin (1000 IU/kg i.p.). Hearts were excised and retrograde perfused via the aorta at a constant pressure of 100 mm Hg, with modified Krebs-Henseleit buffer (pH 7.35–7.45 at 37°C) containing 115 mM NaCl, 4 mM KCl, 1.2 mM MgSO4, 0.9 mM KH2PO4, 22.5 mM NaHCO3, 2.5 mM CaCl2, and 5.5 mM glucose, and oxygenated with 95% O2/5% CO2. Left ventricular developed pressure was measured with a balloon inserted into the left ventricle. Hearts were allocated into three experimental groups: Time control, hearts were buffer perfused for 40 min without ischemia; Ischemia, hearts were equilibrated for 15 min followed by 25 min stop-flow global ischemia at 37°C; Ischemia-reperfusion, hearts were equilibrated for 15 min followed by 25 min stop-flow global ischemia at 37°C and 30 min reperfusion. Panel A: There are no differences in left ventricular developed pressure (LVDP) before ischemia among groups. The LVDP is significantly decreased in hearts following ischemia-reperfusion. Panel B: There are no differences in left ventricular end diastolic pressure (LVEDP) before ischemia among groups, but the LVEDP is elevated at the end of ischemia and during reperfusion. Panel C: The rate of oxidative phosphorylation is decreased in mitochondria following ischemia or ischemia-reperfusion compared to time control when glutamate, succinate, and TMPD-ascorbate are used as complex I, II, and IV substrates, respectively. Panel D: Ischemia decreased CRC compared to time control. Reperfusion further decreased the CRC compared to ischemia, indicating that reperfusion further sensitizes the MPTP opening (Mean ± SEM; *p<0.05 vs. time control; † p<0.05 vs. ischemia). (TIF) [file pone.0118834.s002.tif]

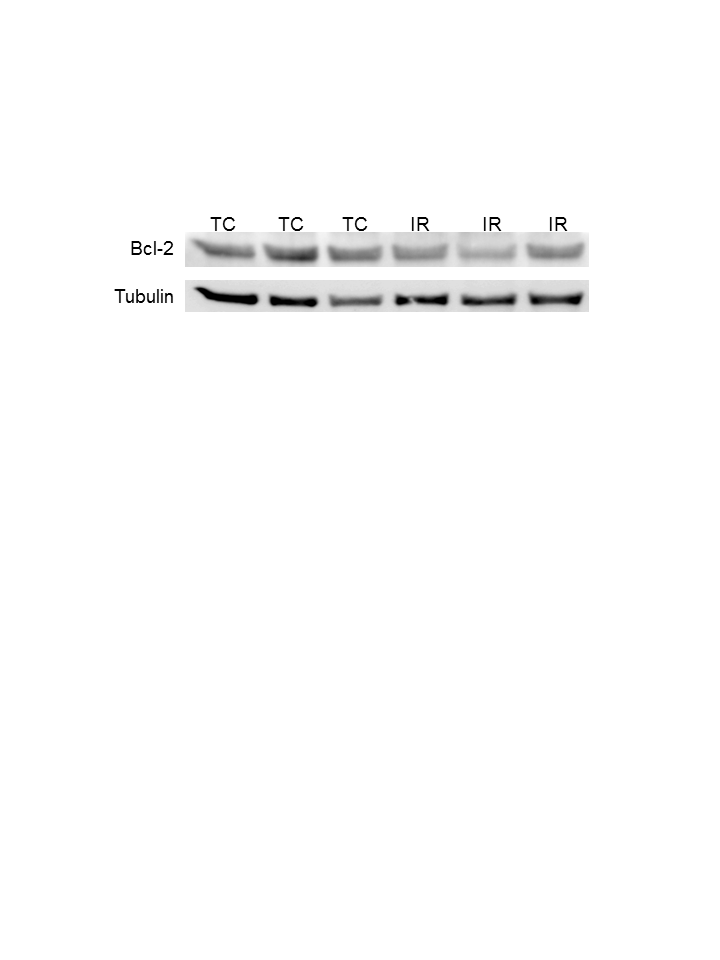

Supplement: S3 Fig — Buffer perfused rat hearts were subjected to 25 min global ischemia and 30 min reperfusion. Bcl-2 content in cytosol was determined using western blotting. The bcl-2 content is decreased in hearts following ischemia-reperfusion compared to time control. Tubulin was used as a protein loading control. (TIF) [file pone.0118834.s003.tif]

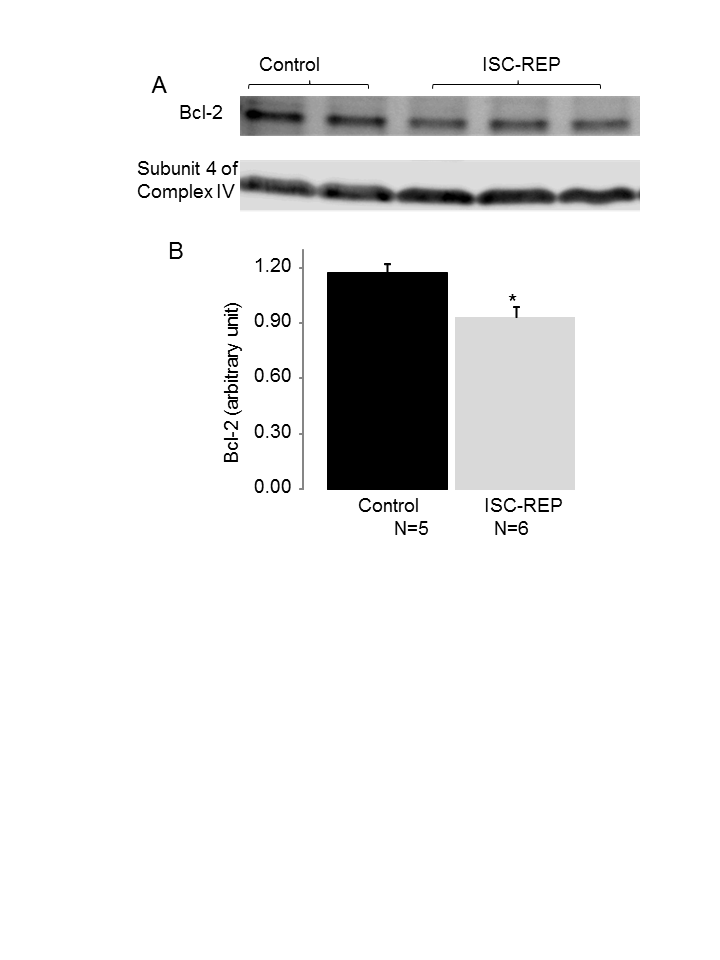

Supplement: S4 Fig — Rat SSM were isolated at the end of heart perfusion. Bcl-2 content was determined using western blotting. Panels A and B: The bcl-2 content is decreased in rat SSM following reperfusion compared to time control (Mean ± SEM; *p<0.05 vs. non-ischemic control). (TIF) [file pone.0118834.s004.tif]
