# Supplementary material for: Inhibition of Bcl-2 Sensitizes Mitochondrial Permeability Transition Pore (MPTP) Opening in Ischemia-Damaged Mitochondria
Source: PLoS One. 2015 Mar 10;10(3):e0118834. doi: 10.1371/journal.pone.0118834 (PMC4354902; doi:10.1371/journal.pone.0118834)
Supplement: S1 Table — (DOCX) [file pone.0118834.s005.docx]

**Table S1. The alteration of oxidative phosphorylation in buffer perfused rat hearts following ischemia (ISC) and reperfusion (REP)**

Figure 3

|  | Glutamate | | | Succinate + rotenone | | |
| --- | --- | --- | --- | --- | --- | --- |
|  | State 3 | State 4 | RCR | State 3 | State 4 | RCR |
| TC (n=5) | 184 ± 14 | 22 ± 3 | 9.23 ± 1.57 | 228 ± 27 | 74 ± 7 | 3.10 ± 0.27 |
| ISC (n=4) | 111 ± 16* | 61 ± 6* | 1.84 ± 0.18* | 98 ± 17* | 64 ± 8 | 1.52 ± 0.07* |
| ISC-REP (n=7) | 86 ± 5* | 47 ± 4* | 1.91 ± 0.21* | 108 ± 8* | 72 ± 2 | 1.49 ± 0.09* |

Mean ± SEM. *p<0.05 vs. time control (TC). RCR, Respiratory control ratio. Glutamate and Succinate + rotenone were used as complex I and complex II substrates, respectively.
