# Supplementary material for: Inhibition of Bcl-2 Sensitizes Mitochondrial Permeability Transition Pore (MPTP) Opening in Ischemia-Damaged Mitochondria
Source: PLoS One. 2015 Mar 10;10(3):e0118834. doi: 10.1371/journal.pone.0118834 (PMC4354902; doi:10.1371/journal.pone.0118834)
Supplement: S2 Table — (DOCX) [file pone.0118834.s006.docx]

**Table S2.** **Ischemia (ISC) alone leads to decreased rate of oxidative phosphorylation in rabbit heart mitochondria**

|  | Glutamate | | | Succinate + rotenone | | |
| --- | --- | --- | --- | --- | --- | --- |
|  | State 3 | State 4 | RCR | State 3 | State 4 | RCR |
| TC (n=4) | 161 ± 6 | 10 ± 1 | 18.9 ± 2.8 | 165 ± 6 | 56 ± 1 | 3.0 ± 0.1 |
| ISC (n=4) | 109 ± 6* | 9 ± 1 | 14.7 ± 2.5 | 127 ± 6* | 46 ± 3* | 2.8 ± 0.1 |

Mean ± SEM. *p<0.05 vs. time control (TC). RCR, Respiratory control ratio.
